# Supplementary material for: Disruption of Osteoprotegerin has complex effects on medial destruction and adventitial fibrosis during mouse abdominal aortic aneurysm formation
Source: PLoS One. 2020 Jul 2;15(7):e0235553. doi: 10.1371/journal.pone.0235553 (PMC7331998; doi:10.1371/journal.pone.0235553)

SUPPLEMENTAL INFORMATION

Supplemental figure 1. Definition of SRA and border for measurements.

(A) Representative images of aortas from AngII-infused mice. The suprarenal aorta (SRA) is indicated proximal region of the both right and left renal arteries. The red line indicates the maximum diameter used to measure the external diameter of the SRA. Scale bars indicate 1 mm. **(B)** Representative magnified EVG-stained image of the aorta. The border of the aortic lumen is marked by a white dotted circle, which was also used to measure the internal area of the SRA. Red lines indicate measurements of the width of the medial layer (m) on the side undisrupted by atherosclerotic plaques (p). **(C)** Representative magnified AZAN-stained image of the aorta. Outer and inner borders of the adventitial layer and hematoma (h) are marked by white dotted circles, which were used to measure and calculate the relative area of the adventitia. Scale bars indicate 100 μm. The aortic lumen, medial layer, adventitial layer, and surrounding fat tissue are marked as l, m, a, and f, respectively.

Supplemental figure 2. Data for H_2O-infused control group.

(A) Aortas of H_2O-infused *ApoE*$^{-/-}$*Opg*$^{+/+}$ and *ApoE*$^{-/-}$*Opg*$^{-/-}$ mice. Scale bars indicate 1 mm. **(B)** Representative images of aortas in the H_2O-infused group stained with HE, EVG, and AZAN. Areas selected by boxes with black dotted lines are magnified in the lower panels. Scale bars indicate 100 μm. **(C-E)** External diameters **(C)** and internal areas **(D)** of SRAs, and relative areas of adventitias **(E)** in H_2O-infused *ApoE*$^{-/-}$*Opg*$^{+/+}$ (dark gray, n=4) and *ApoE*$^{-/-}$*Opg*$^{-/-}$ (bright gray, n=5) mice. **(F)** Survival rate at 0 and 28 days after AngII infusion. **(H)** Total cholesterol concentration (mg/dL) in serum at 0 and 28 days after AngII infusion, n=3. N.S.: not significant.

Supplemental figure 3. Aneurysm diameter measurements

(A) Images of cross-sections of aortas of AngII-infused mice stained with EVG. Scale bars indicate 0.1 mm. **(B)** Images of cross-sections of aortas of AngII-infused mice stained with AZAN.

Supplemental figure 4. AngII infusion leads to collagen accumulation in aortas of *ApoE$^{-/-}$Opg$^{-/-}$* mice

(A) Immunofluorescence images of aortic sections stained with an anti-collagen I antibody. Scale bars indicate 100 μm. **(B)** Immunofluorescence images of aortic sections stained with an anti-collagen III antibody. Scale bars indicate 100 μm. Skin was used as a positive control. Areas selected by the box with dotted lines on the skin are magnified below. **(C)** Percent area of collagen I expression in aortas of H_2O-infused *ApoE$^{-/-}$Opg$^{+/+}$* (n=4) and *ApoE$^{-/-}$Opg$^{-/-}$* (n=5) mice. N.S.: not significant.

Supplemental figure 5. Mmp and Trail expression following AngII or H_2O infusion

(A) Immunofluorescence images of aortic sections stained with anti-Mmp9 antibody from AngII-infused mice. **(B-C)** *Mmp9* **(B)** and *Mmp2* **(C)** mRNA expression in SRAs at days 0, 7, and 28 after initiation of AngII infusion in *ApoE$^{-/-}$Opg$^{+/+}$* (n=4, 5, 5) and *ApoE$^{-/-}$Opg$^{-/-}$* (n=5, 5, 5) mice. **(D)** Immunofluorescence images of aortic sections stained with anti-collagen I, -SMA, -vimentin, -Ki67, -collagen III, -Trail -Mmp-9, and F4/80 antibodies from H_2O-infused mice. Scale bars indicate 100 μm. **(E-F)** Percent area of SMA **(E)** and Trail **(F)** expression in aortas of H_2O-infused *ApoE$^{-/-}$Opg$^{+/+}$* (n=4) and *ApoE$^{-/-}$Opg$^{-/-}$* (n=5) mice. N.S.: not significant. **(G)**

Immunofluorescence images of aortic sections stained with an anti-F4/80 antibody from AngII-infused *ApoE*$^{-/-}$*Opg*$^{+/+}$ and *ApoE*$^{-/-}$*Opg*$^{-/-}$ mice.

Supplemental figure 6. Schematic model of changes in aortic tissue with or without *Opg* in the AngII-induced *ApoE*-KO mouse model

Administration of AngII in the *ApoE*-KO mouse model leads to inflammation, destruction of aortic tissue, and expression of inflammatory cytokines. In *Opg*-deficient mice, AngII induces the expression of Trail, which may stimulate the appearance, proliferation, and migration of myofibroblasts, as indicated in previous reports [1, 2]. This accumulation of myofibroblasts may result in adventitial thickening with fibrosis, e.g., as a result of collagen I deposition. A substantial amount of collagen I in the adventitia could suppress dilatation of the inner diameter of the suprarenal aorta (SRA) by maintaining the stiffness and structural integrity of the lumen [3, 4]. Thus, the characteristic changes in the adventitia of *ApoE*$^{-/-}$*Opg*$^{-/-}$ mice, including the accumulation of myofibroblasts and collagen I, may underlie the suppression of aneurysm formation and obscure the influence of tissue destruction.

<References>

1. Bumdelger B, Kokubo H, Kamata R, Fujii M, Yoshimura K, Aoki H, et al. Osteoprotegerin Prevents Development of Abdominal Aortic Aneurysms. PLoS One. 11(1):e0147088. Epub 2016/01/20. doi: 10.1371/journal.pone.0147088
PONE-D-15-23302 [pii]. PubMed PMID: 26783750.

2. Kavurma MM, Schoppet M, Bobryshev YV, Khachigian LM, Bennett MR. TRAIL stimulates proliferation of vascular smooth muscle cells via activation of NF-kappaB and induction of insulin-like growth factor-1 receptor. J Biol Chem. 2008;283(12):7754-62. Epub 2008/01/08. doi: M706927200 [pii]
10.1074/jbc.M706927200. PubMed PMID: 18178561.

3. Dobrin PB, Mrkvicka R. Failure of elastin or collagen as possible critical connective tissue alterations underlying aneurysmal dilatation. Cardiovasc Surg. 1994;2(4):484-8. Epub 1994/08/01. PubMed PMID: 7953454.

4. Malfait F. Vascular aspects of the Ehlers-Danlos Syndromes. Matrix Biol. 71-72:380-95. Epub 2018/05/02. doi: S0945-053X(18)30072-6 [pii]
10.1016/j.matbio.2018.04.013. PubMed PMID: 29709596.

Bumdelger et al.
Supplemental figure 1

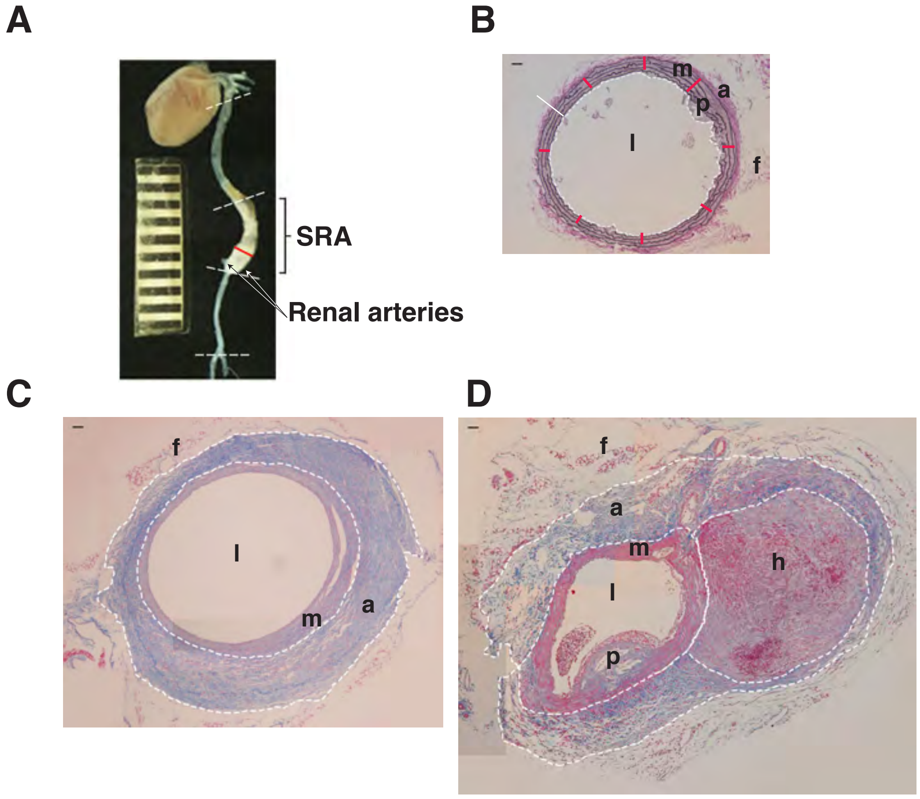

Bumdelger et al.
Supplemental figure 2

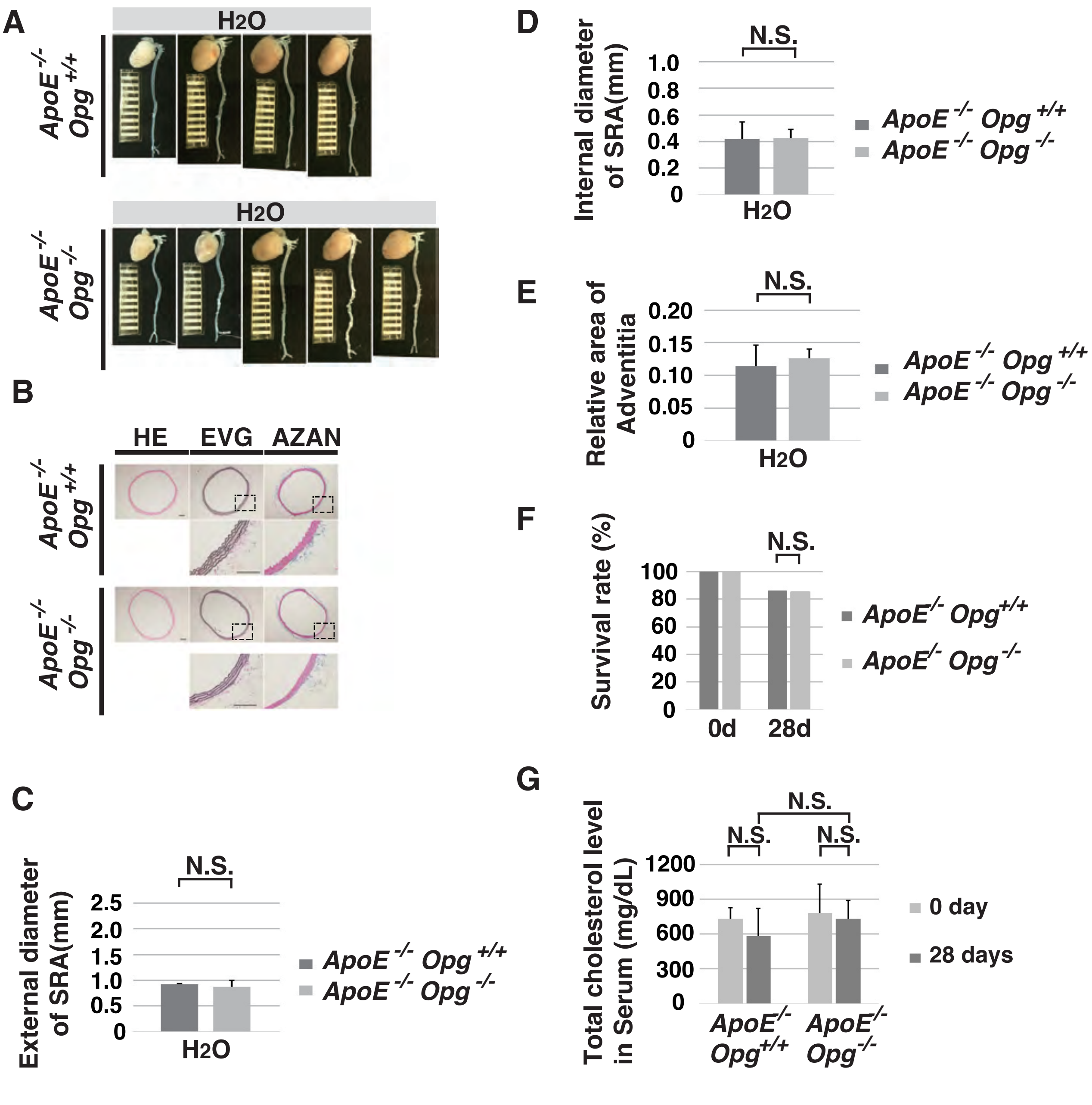

Bumdelger et al.
Supplemental figure 3

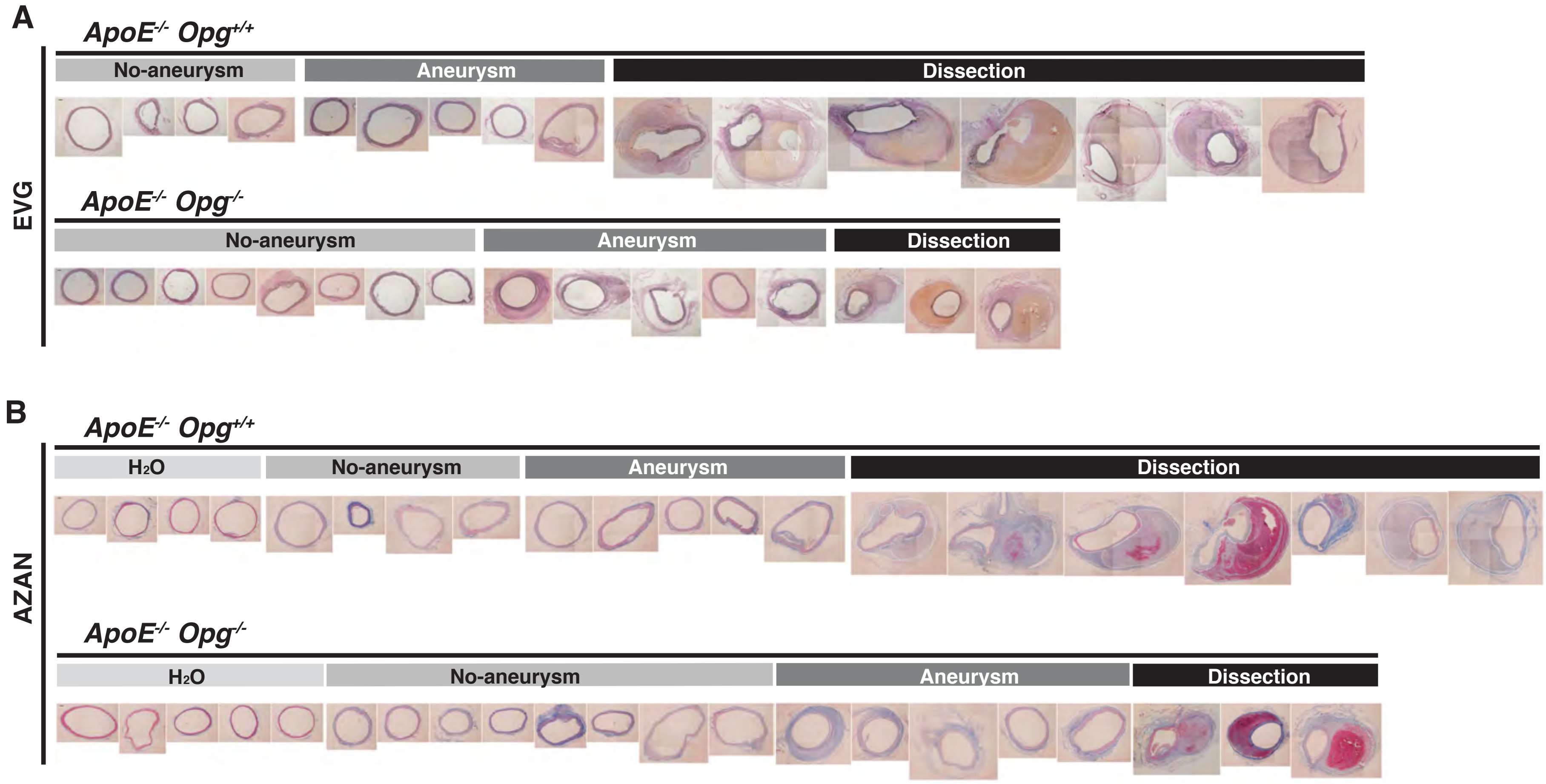

Bumdelger et al.
Supplemental figure 4

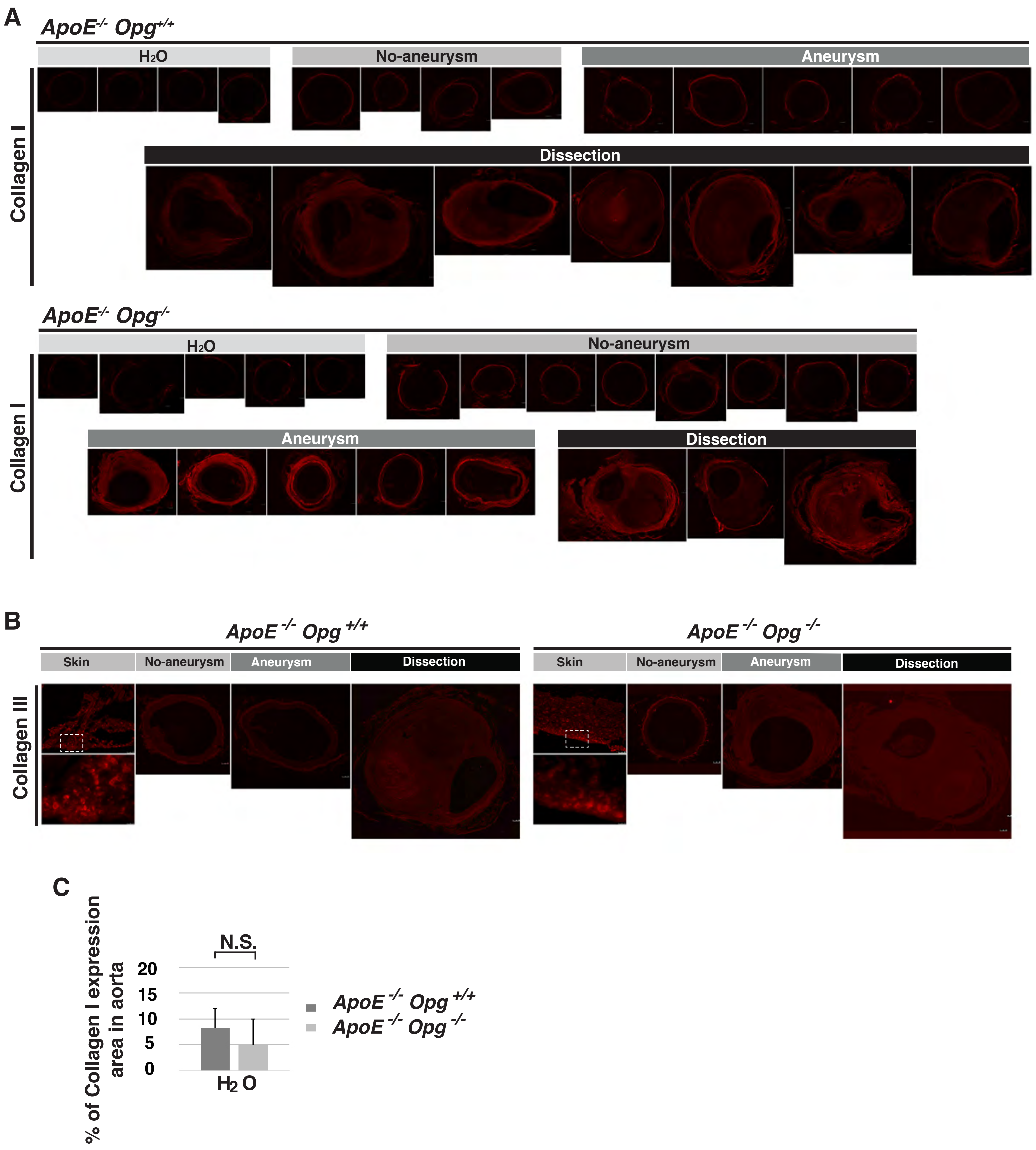

Bumdelger et al.
Supplemental figure 5

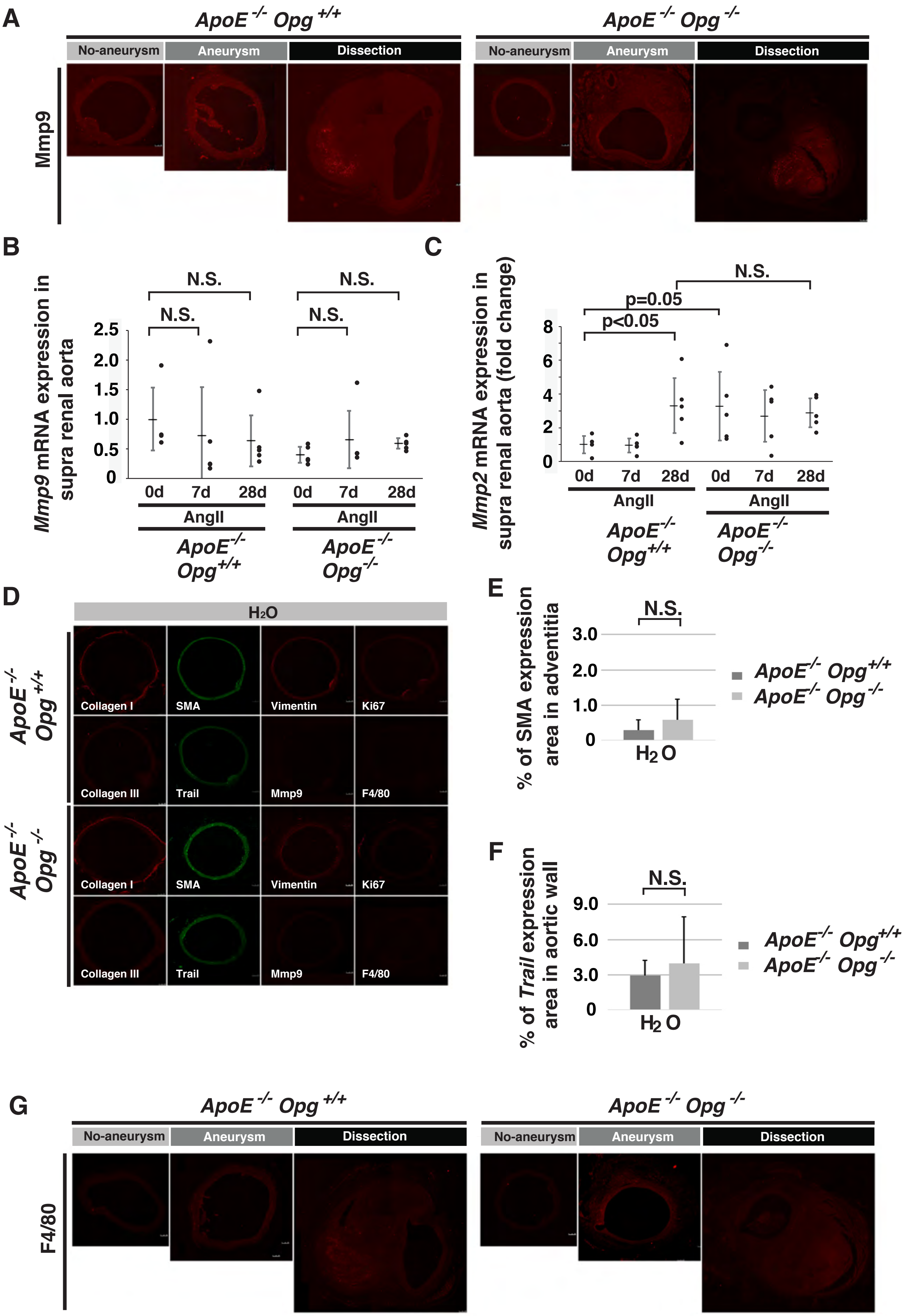

Bumdelger et al.
Supplemental figure 6

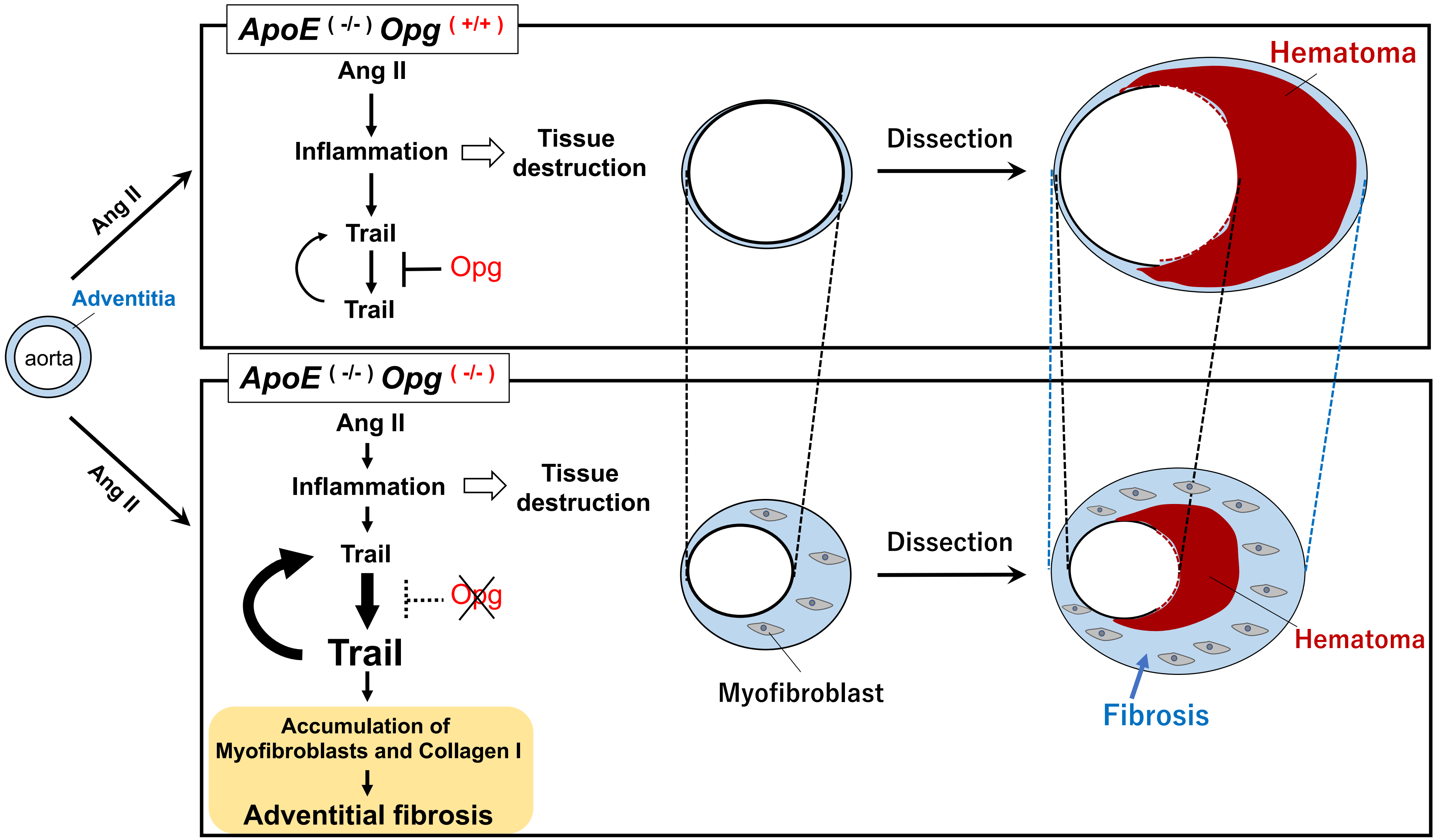

Supplement: S1 File — (PDF) [file pone.0235553.s001.pdf]
