# Supplementary material for: Disruption of Osteoprotegerin has complex effects on medial destruction and adventitial fibrosis during mouse abdominal aortic aneurysm formation
Source: PLoS One. 2020 Jul 2;15(7):e0235553. doi: 10.1371/journal.pone.0235553 (PMC7331998; doi:10.1371/journal.pone.0235553)
Supplement: Attachment — Submitted filename: Response_to reviewer.docx [file pone.0235553.s002.docx]

Reviewer #1: Main summary
*In the paper by Bumdelger et al, the authors evaluate the role of aneurysm/dissection in the Apoe-/- osteoprotegerin (Opg)-knockout mouse model. Interestingly, the authors introduce that using the calcium-chloride model, that deficiency of Opg had larger aneurysms and increased expression of matrix metalloproteinase-9 (MMP9) and TNF-related apoptosis-inducing ligand (TRAIL). Within this paper, using 7-month-old mice, AngII-infusion into Apoe-/- Opg-/- male mice resulted in lower external abdominal aortic diameter and lower incidence of aneurysms and dissections. The group goes on to do histological analysis and finds that collagen I expression is higher in the Apoe-/- Opg-/- mice under AngII-infusion, however this did not occur under vehicle conditions (water-infused, Supplemental Fig. 3). The group looks at alpha-smooth muscle actin, vimentin, TRAIL, F4/80, and collagen I and find that within mice that experience an aneurysm, Apoe-/- Opg-/- have increased expression of actin, vimentin, TRAIL, and collagen I that results in fibrotic remodeling that may help to lower the possibility of developing a dissection or aortic rupture. While the studies are interesting, there are some details that need to be addressed for these experiments.*

Major concerns
*1. If I understand correctly, both with calcium-chloride and the AngII-infusion model, TRAIL levels go up significantly. The authors state that TRAIL goes up, because the decoy receptor (Opg) is no longer expressed. If this is the case, then immunostaining for TRAIL should also be increased in the water-infused mice. If TRAIL expression is only tied to Opg, then the reader would benefit from knowing this in the water-infused mice. This could be placed in the supplement and help with the overall understanding of TRAIL within these models.*

We thank the Reviewer for the suggestion. We did not detect significant differences in Trail expression between H_2_O-infused *ApoE^-/-^Opg*^+/+^ and *ApoE^-/-^Opg^-/-^* mice. However, AngII infusion significantly increased Trail expression only in *ApoE^-/-^Opg^-/-^* mice. These data were added to Supplemental Figures 5D and 5F.

2. *The authors do a good job of separating out non-aneurysm from aneurysm and dissection. In Figures 1-3, the authors examine all disease types, however in Figure 4, only aneurysm tissue is examined. Is it possible to look at non-aneurysm and dissection tissues for alpha-actin, vimentin, F4/80 and collagen I? It might help to keep it all consistent from figure to figure. There was also quantification for Figure 3, but none for Figure 4. Is it possible to do this for only 1 of the 4 proteins observed? Maybe vimentin or smooth muscle actin since quantification has already been done for collagen I.*

We thank the Reviewer for the suggestion. As suggested, we performed IHC studies using antibodies against α-SMA, vimentin, and F4/80 for all disease types and also quantified the α-SMA expressing area in the adventitia. These data were added to Figure 4 (former Figure 4 has been renumbered to be Figure 5). Corresponding changes were also made to the manuscript (page 7, lines 18-19).

3. *Have the authors evaluated collagen IV levels in the aortic basal lamina? Is it increased in the Opg-knockout similar to collagen I?*

Unfortunately, we did not perform that experiment. We did, however, perform IHC studies using an anti-collagen III antibody and found that collagen III was not expressed in the vascular wall of both *ApoE^-/-^Opg*^+/+^ and *ApoE^-/-^Opg^-/-^* mice. These data were added to Supplemental Figure 4B.

4. *It might be interesting to look at MMP2 and MMP9 levels in these Opg-knockout mice. My guess is that this might be lower in the Opg-knockout mouse, but if there is excessive remodeling off the tissue, then maybe not? This could be explored at the mRNA level if needed. Methods indicate that mRNA was collected, however I could not find any data concerning this.*

We performed quantitative RT-PCR to measure *Mmp2* and *Mmp9* transcript levels. No significant differences were detected in *Mmp9* levels between *ApoE^-/-^ Opg^+/+^* and *ApoE^-/-^ Opg^-/-^* mice, although *Mmp2* levels were elevated in *ApoE^-/-^ Opg^-/-^* mice. Interestingly, AngII infusion did not influence *Mmp2* levels, implying that either Mmp2 or Mmp9 is not involved in the histological changes induced by AngII infusion. These data were included in Supplemental Figures 5A-5C.

Minor concerns
*1. Was any ultrasound data collected? If it was collected, then placing this within the manuscript would benefit the reader.*

We appreciate the Reviewer’s suggestion. However, we unfortunately did not collect ultrasound data.

2. *Methods state that the mice were aged out to 6 months, however abstract indicates 7 months. Which is correct?*

We thank the Reviewer for pointing this out. The manuscript was revised to clarify that AngII infusions started when mice were 6 months of age. Mice were sacrificed at 7 months of age.

3. *Suprarenal aorta (SRA) appears in the Introduction first and should be defined there.*

Suprarenal aorta (SRA) is defined in the manuscript on page 5 (line 5). We also added a representative image of the aorta to help explain the portion to which the SRA corresponds (Supplemental Figure 1).

4. *“Revealed” is misspelled in the Introduction.*

This typographical error was corrected, accordingly (page 4, line 9).

5. Statistical analysis should be done on the incidence data in Figure 1.

Statistical analysis was performed for Figure 1C. We used ordinal logistic regression analysis.

6. *Did the authors measure any plasma/serum measures in the mice. Since these mice would be hypercholesterolemic, did the authors look at serum cholesterol levels?*

Cholesterol concentrations were available and added to Supplemental Figure 2G.

7. *Did the authors look at TGF-beta or FGF in the aorta? Since the authors conclude that fibrosis remodeling has occurred in the Opg-knockout, then measurement of these factors could help to enhance the overall conclusion.*

We thank the Reviewer for the helpful suggestion, and agree that Tgf-β may have an effect on fibrosis remodeling. We measured *Tgf-β1* levels by RT-PCR and found that they increased after AngII infusion, consistent with a role for Tgf-β1 in enhancing fibrosis remodeling. We added an explanation of this to the manuscript (page 7, lines 10-13), and included the data in Figure 3C.

8. *There doesn’t seem to be any rupture information within these studies. Can the authors report on how many mice were lost due to aortic rupture in the wildtype and knockout mice?*

Unfortunately, we only counted the number of dead mice and did not confirm whether their deaths were due to aortic rupture. Supplemental Figure 2F provides the survival data.

Reviewer #2: The paper entitled "Disruption of Osteoprotegerin has complex effects on medial destruction and adventitial fibrosis during mouse abdominal aortic aneurysm formation" by Bumdelger et al examined the effect of osteoprotegerin (Opg) in abdominal aneurysm. While there is already conflicting data published on the role of Opg on aneurysm: one by the same authors concluding preventive role of Opg in CaCl2-induced AAA and another by Moran et al 2014 concluding disease promoting role of Opg in AngII-induced AAA; this paper attempts to confirm the role of Opg in AngII-induced mouse model of AAA. However, at current state of the paper, many conclusions are not fully supported by the data provided. I have following concerns:

1) *Figure 1: The title of Figure 1 and conclusion "Opg deficiency limits AngII-induced aortic dissection and dilatation" is overstated. External diameter is not significantly reduced with Opg deficiency. How do the authors define dissection? The authors need to classify aneurysm based on Daugherty's classification (PMID: 11606327). Were they abdominal or thoracic dissection? The data is not statistically analyzed to say preventive effect of Opg deficiency on aortic dissection. What about the mortality rates and mortality data in these experimental mice?*

We thank the Reviewer for these insightful comments. As suggested, we revised the title and first paragraph of the Results section to be more commensurate in scope with the data.

We did not initially follow Daugherty’s classification, since Moran’s group did not use that classification system. Nonetheless, we newly evaluated aneurysms based on the presence or absence of hematoma by morphological observation, similar to Daugherty’s classification. We cite Daugherty’s article and clarify that our classification method is a modified version of Daugherty’s method (page 5, lines 14-17). Our focus was mainly abdominal dissection at the SRA, although dissection was also observed in the thoracic region.

As mentioned by Reviewer #1, we statistically analyzed the incidence rate of three disease phases using ordinal logistic regression. In result, there was a non significant tendency for aneurysm and aortic dissection in *ApoE^-/-^ Opg^-/-^* mice. We included the mortality data in Supplemental Figure 2F.

2) *Figure 2: I could not understand the mechanism behind adventitial thickening and aortic dissection. Do the authors have any speculation on how does the adventitial thickening leads to smaller aortic diameter and dissection in ApoE-/-Opg-/- mice?*

To address this comment, we provide a schematic and description of how we believe adventitial thickening leads to smaller aortic diameter and dissection in *ApoE^-/-^Opg^-/-^* mice (Supplemental Figure 6).

We speculate that administration of AngII in the *ApoE*-KO mouse model leads to inflammation, destruction of aortic tissue, and expression of inflammatory cytokines. In *Opg* deficient mice, AngII induces the expression of Trail, which may stimulate the appearance, proliferation, and migration of myofibroblasts, as indicated in previous reports. This accumulation of myofibroblasts may result in adventitial thickening with fibrosis, e.g., as a result of collagen I deposition. A substantial amount of collagen I in the adventitia could suppress dilatation of the inner diameter of the suprarenal aorta (SRA) by maintaining the stiffness and structural integrity of the lumen (Dobrin PB, et al. PMID: 7953454, Malfait F. PMID: 29709596). Thus, the characteristic changes in the adventitia of *ApoE^-/-^Opg^-/-^* mice, including the accumulation of myofibroblasts and collagen I, may underlie the suppression of aneurysm formation and obscure the influence of tissue destruction. Supporting this potential mechanism is a study by Dobrin et al., which found in ex vivo experiments that additional collagenase, rather than elastase, led to the enlargement of the lumen of blood vessels (Dobrin PB, et al. PMID: 7953454).

3) *Figure 3: What about collagen III? Picrosirius red staining needs to be performed for differential staining of collagen I vs collagen III.*

As suggested, we performed IHC experiments with antibodies against collagen type III. The expression of collagen III was much lower than that of collagen I and was not upregulated in response to AngII stimulation. We confirmed that the anti-collagen III antibody works by using a section of skin tissue as a positive control (Supplemental Figure 4B).

4) *Figure 4. The authors show that cells accumulated in adventitial region of Apoe-/-Opg-/- mice are likely myofibroblasts since they co-express SMA and vimentin. However, the Figure 4I shows more infiltration of F4/80+ macrophages in Apoe-/-Opg-/- compared to Apoe-/- alone in 4F. And it is surprising that there are fewer macrophages and less adventitial thickening in Apoe-/- mice in 4F and 4E. How does increased Trail expression, increased inflammation, myofibroblast accumulation and fibrotic remodeling can be protective to aortic dilation and dissection? The data presented and conclusion do not match. Instead, these data imply that there would be more disease in Apoe-/-Opg-/- mice because of more inflammation.*

We describe the mechanism by which we speculate Trail expression is increased, inflammation is stimulated, myofibroblasts accumulate, and how fibrosis could suppress aortic dilatation and dissection in Supplemental Figure 6.

In further experiments, we detected an increase in macrophages, but no upregulation of Mmps, suggesting that inflammation does not play a major role in the observed histological changes. On the other hand, the thickened adventitia was filled with myofibroblasts and had substantial collagen I accumulation. As mentioned above in response to point #2, the presence of collagenase, rather than elastase, has been shown to cause enlargement of the lumen of blood vessels in a previous ex vivo study (Dobrin PB, et al. PMID: 7953454). These data indicate that fibrotic remodeling can be protective against aortic dilatation and dissection.

5) *Why 6-months (24 weeks) old mice are used for the aneurysm studies? Aneurysm studies are best performed in 8-12 weeks old mice.*

Our initial goal was to reproduce the experiments performed by Moran’s group, and thus matched the ages of mice with their study.

6) *Poor quality of writing: There are several grammatical, typo and spelling errors in the sentences. The paper needs significant English editing.*

We had the manuscript checked and edited by a native English speaker.

Reviewer #3: The paper entitled " Disruption of Osteoprotegerin has complex effects on medial destruction and adventitial fibrosis during mouse abdominal aortic aneurysm formation" by Kokubo et al concluded that fibrotic remodeling of the aorta induced by myofibroblast accumulation might be an important pathological event which limits AngII-induced aortic dilatation in ApoE -/- Opg -/- mice.

The conclusion of the manuscript is not substantially based upon the experimental data and is highly speculative.
Specifically:

1. *The statement ‘Opg deficiency limits AngII-induced aortic dissection and dilatation’ is an overstatement of the data. The AAA definition is missing and also lacks statistical analysis.*

We thank the Reviewer for raising this point. We agree and have revised the manuscript to avoid overstating the present findings (page 5, line 2, and title) and to include the definition of AAA (page 13, lines 17-19).

2. No evidence to show the specificity of antibodies to detect vSMCs and fibroblasts. Single IHC may not be sufficient to draw the conclusions.

In the previous version of the manuscript, myofibroblasts were identified by IHC using anti-α-SMA or anti-vimentin antibodies. However, we agree that single IHC data cannot be used definitively to identify myofibroblasts. Accordingly, we performed immunofluorescence-based double staining with anti-α-SMA and anti-vimentin antibodies. Cells which were positive for both markers were considered to be myofibroblasts, and cells positive only for α-SMA were considered to be vSMCs (Skalli O, et al. PMID: 2918221).

3. *The interpretation that ‘Opg deficiency was found to limit AngII-induced aortic dissection and dilatation’ is not convincing.*

We added a discussion of how we arrived at that interpretation in Supplemental Figure 6 (and its accompanying legend). As mentioned in our reply to a similar comment from Reviewer #2, we believe that adventitial thickening accompanying collagen I accumulation has a preventive effect on enlargement of the aortic lumen and total outer diameter. According to a previous study, it was found in ex vivo experiments that additional collagenase, rather than elastase, led to the enlargement of the lumen of blood vessels (Dobrin PB, et al. PMID: 7953454). When this finding is applied to the present context, the accumulation of collagen I could be one of the causes underlying the preventive effect on enlargement of the aortic lumen.

4. *There are no reports/data to show that fibrotic remodeling of the aorta might protects against AAA.*

We agree with the Reviewer that there is no publication directly on point. However, as mentioned in the reply to the previous comment, Dobrin et al. found that a reduction in collagen I decreases the stiffness of the vascular wall (Dobrin PB, et al. PMID: 7953454). Moreover, according to a study by Malfait et al., corruption of collagen promoted the formation of aneurysms and dissection (Malfait F. et al. PMID: 29709596). Taken together, we believe our speculation that collagen I accumulation in the adventitia results in increased stiffness of the vascular wall to be reasonable.

5. *Why the authors used 6 month old mice?*

Our initial goal was to reproduce the experiments conducted by Moran’s group. Accordingly, we used the experimental conditions described in their paper, including the age of mice.

6. *Since aortic dissection in these mice occur at early stage of the disease (4-10 days), determination of visible blood at day 28 may not be a good indicator for aortic dissection.*

As mentioned above, our initial goal was to reproduce the experiments conducted by Moran’s group. To this end, we mirrored the conditions they used, including those for observation period (i.e., 28 days after AngII infusion). Accordingly, we did not perform earlier stage observations. Classification of aneurysms by Daugherty’s group was also performed 28 days after AngII infusion, which is when the severity of aneurysms shows diversity in this AAA model (Daugherty A, et al. PMID: 11606327). Based on the above, we believe 28 days to be appropriate for observing the phenotype of mice.

7. *Please check for grammatical errors.*

 We had the manuscript checked and edited by a native English speaker.
