# Supplementary material for: Disruption of Osteoprotegerin has complex effects on medial destruction and adventitial fibrosis during mouse abdominal aortic aneurysm formation
Source: PLoS One. 2020 Jul 2;15(7):e0235553. doi: 10.1371/journal.pone.0235553 (PMC7331998; doi:10.1371/journal.pone.0235553)
Supplement: Attachment — Submitted filename: response to reviewer-re.docx [file pone.0235553.s003.docx]

Re: PONE-D-20-04332R1

Additional Editor Comments (if provided):

Please provide tables in the supplementary material with details on the antibodies (vendor, cat#, lot#, specificity , literature citation using the same antibody) and primers (sequence, annealing temperature, PCR product size) used for the study


[Note: HTML markup is below. Please do not edit.]

Reviewers' comments:

Reviewer's Responses to Questions

**Comments to the Author**

1. If the authors have adequately addressed your comments raised in a previous round of review and you feel that this manuscript is now acceptable for publication, you may indicate that here to bypass the “Comments to the Author” section, enter your conflict of interest statement in the “Confidential to Editor” section, and submit your "Accept" recommendation.

Reviewer #1: (No Response)

Reviewer #2: All comments have been addressed

Reviewer #3: All comments have been addressed

2. Is the manuscript technically sound, and do the data support the conclusions?

The manuscript must describe a technically sound piece of scientific research with data that supports the conclusions. Experiments must have been conducted rigorously, with appropriate controls, replication, and sample sizes. The conclusions must be drawn appropriately based on the data presented. 

Reviewer #1: Yes

Reviewer #2: Yes

Reviewer #3: Yes

3. Has the statistical analysis been performed appropriately and rigorously? 

Reviewer #1: Yes

Reviewer #2: Yes

Reviewer #3: N/A

4. Have the authors made all data underlying the findings in their manuscript fully available?

The [PLOS Data policy](https://jpn01.safelinks.protection.outlook.com/?url=http%3A%2F%2Fwww.plosone.org%2Fstatic%2Fpolicies.action%23sharing&data=02%7C01%7Chkokubo%40hiroshima-u.ac.jp%7C8d4ed53d7c8e48884f8d08d80bfa31a5%7Cc40454ddb2634926868d8e12640d3750%7C1%7C0%7C637272516486505608&sdata=YBoerFOqT%2F%2BwYTqgZHSRUCpOWtzObc4DuQDAgJdo%2B6Q%3D&reserved=0) requires authors to make all data underlying the findings described in their manuscript fully available without restriction, with rare exception (please refer to the Data Availability Statement in the manuscript PDF file). The data should be provided as part of the manuscript or its supporting information, or deposited to a public repository. For example, in addition to summary statistics, the data points behind means, medians and variance measures should be available. If there are restrictions on publicly sharing data—e.g. participant privacy or use of data from a third party—those must be specified.

Reviewer #1: Yes

Reviewer #2: Yes

Reviewer #3: Yes

5. Is the manuscript presented in an intelligible fashion and written in standard English?

PLOS ONE does not copyedit accepted manuscripts, so the language in submitted articles must be clear, correct, and unambiguous. Any typographical or grammatical errors should be corrected at revision, so please note any specific errors here.

Reviewer #1: Yes

Reviewer #2: Yes

Reviewer #3: Yes

6. Review Comments to the Author

Please use the space provided to explain your answers to the questions above. You may also include additional comments for the author, including concerns about dual publication, research ethics, or publication ethics. (Please upload your review as an attachment if it exceeds 20,000 characters)

*Reviewer #1: Please make the following small editorial changes.
1. Hematoma is misspelled on page 13, second to last line.*

This typographical error was corrected accordingly.

*2. Catalog numbers for all antibodies should be included to help with replication of studies.*

We added a table in the manuscript to show catalog numbers for all antibodies followed by reviewer’s suggestion. We also changed text to cite this table in the section fo materials and methods, pp15 line 5-**6**.

*3. Need a hyphen for alpha-SMA in Figure 4 legend*

We deleted α in the Figure 4 legend (page 23) because we have defined the α-smooth muscle actin as SMA in page 4, line 14.

*4. Need MMP2 primer sequence in the Methods section.*

The MMP2 primer sequence has already been added in the method section (page 15, last line). To avoid confusion, we added an additional table in the manuscript to provide details on primers we used. We also changed text to cite this table in the section fo materials and methods, pp15 line 15.

*5. Should state both "MMP-2 and MMP-9" in Discussion section, page 10, second to last line.*

As followed reviewer’s suggestion, we stated both MMP-9 and MMP-2.

Reviewer #2: All the comments have been nicely addressed by the authors with addition of data. I have no further questions.

Reviewer #3: All the comments have been addressed adequately by the authors. The reviewer has no more major concerns.

7. PLOS authors have the option to publish the peer review history of their article ([what does this mean?](https://jpn01.safelinks.protection.outlook.com/?url=https%3A%2F%2Fjournals.plos.org%2Fplosone%2Fs%2Feditorial-and-peer-review-process%23loc-peer-review-history&data=02%7C01%7Chkokubo%40hiroshima-u.ac.jp%7C8d4ed53d7c8e48884f8d08d80bfa31a5%7Cc40454ddb2634926868d8e12640d3750%7C1%7C0%7C637272516486505608&sdata=M2uo2K%2FLpC8MoShM9GPB1pqs03yhaL533yOLJ%2FYHqTk%3D&reserved=0)). If published, this will include your full peer review and any attached files.


If you choose “no”, your identity will remain anonymous but your review may still be made public.


**Do you want your identity to be public for this peer review?** For information about this choice, including consent withdrawal, please see our [Privacy Policy](https://jpn01.safelinks.protection.outlook.com/?url=https%3A%2F%2Fwww.plos.org%2Fprivacy-policy&data=02%7C01%7Chkokubo%40hiroshima-u.ac.jp%7C8d4ed53d7c8e48884f8d08d80bfa31a5%7Cc40454ddb2634926868d8e12640d3750%7C1%7C0%7C637272516486505608&sdata=fMN0DBfK77ozAlzXCXUCLnWwVFGUHQEksyuBOlvYlqo%3D&reserved=0).

Reviewer #1: No

Reviewer #2: No

Reviewer #3: Yes: Chetan P Hans


[NOTE: If reviewer comments were submitted as an attachment file, they will be attached to this email and accessible via the submission site. Please log into your account, locate the manuscript record, and check for the action link "View Attachments". If this link does not appear, there are no attachment files.]

While revising your submission, please upload your figure files to the Preflight Analysis and Conversion Engine (PACE) digital diagnostic tool, [https://pacev2.apexcovantage.com/](https://jpn01.safelinks.protection.outlook.com/?url=https%3A%2F%2Fpacev2.apexcovantage.com%2F&data=02%7C01%7Chkokubo%40hiroshima-u.ac.jp%7C8d4ed53d7c8e48884f8d08d80bfa31a5%7Cc40454ddb2634926868d8e12640d3750%7C1%7C0%7C637272516486515599&sdata=8O9Po0siGOf74iI1JTCTLt%2BpEkccXcMsBL%2BvKIwDig0%3D&reserved=0). PACE helps ensure that figures meet PLOS requirements. To use PACE, you must first register as a user. Registration is free. Then, login and navigate to the UPLOAD tab, where you will find detailed instructions on how to use the tool. If you encounter any issues or have any questions when using PACE, please email PLOS at [figures@plos.org](mailto:figures@plos.org). Please note that Supporting Information files do not need this step.
